# Comparative analysis of chloroplast and mitochondrial genomes of sweet potato provides evidence of gene transfer

**DOI:** 10.1038/s41598-024-55150-1

**Published:** 2024-02-24

**Authors:** GuoLiang Li, Hong Zhang, Zhaomiao Lin, Huawei Li, Guochun Xu, Yongqing Xu, Rongchang Ji, Wenbin Luo, Yongxiang Qiu, Sixin Qiu, Hao Tang

**Affiliations:** Institute of Crop Sciences, Fujian Academy of Agricultural Sciences, Fuzhou, Fujian China

**Keywords:** Evolution, Molecular evolution

## Abstract

The increasing number of plant mitochondrial DNA genomes (mtDNA) sequenced reveals the extent of transfer from both chloroplast DNA genomes (cpDNA) and nuclear DNA genomes (nDNA). This study created a library and assembled the chloroplast and mitochondrial genomes of the leafy sweet potato better to understand the extent of mitochondrial and chloroplast gene transfer. The full-length chloroplast genome of the leafy sweet potato (OM808940) is 161,387 bp, with 132 genes annotated, including 87 protein-coding genes, 8 rRNA genes, and 37 tRNA genes. The mitochondrial genome (OM808941) was 269,578 bp in length and contained 69 functional genes, including 39 protein-coding genes, 6 rRNA genes, and 24 tRNA genes. 68 SSR loci were found in the leafy sweet potato organelle genome, including 54 in the chloroplast genome and 14 in the mitochondria genome. In the sweet potato mitochondrial genome, most genes have RNA editing sites, and the conversion ratio from hydrophilic amino acids to hydrophobic amino acids is the highest, reaching 47.12%. Horizontal transfer occurs in the sweet potato organelle genome and nuclear genome. 40 mitochondrial genome segments share high homology with 14 chloroplast genome segments, 33 of which may be derived from chloroplast genome horizontal transfer. 171 mitochondrial genome sequences come from the horizontal transfer of nuclear genome. The phylogenetic analysis of organelle genes revealed that the leafy sweet potato was closely related to the tetraploid wild species *Ipomoea tabascana* and the wild diploid species *Ipomoea trifida*.

## Introduction

In recent years, with the gradual deepening of genome research, gene transfer between different genomes (mitochondrial, nuclear and chloroplast) in the plant has been uncovered^1^. Horizontal gene transfer (HGT) may have been more common in the early evolution of unicellular eukaryotes and less common in multicellular eukaryotes, owing to the numerous barriers required for transport across the nuclear envelope. Moreover, the endogenous transfer of plant mitochondrial genes into the nucleus is a continuous process. During the evolution of plant mitochondrial proteome, many primitive functions have been lost or reconfigured in the nucleus. By sequencing the genome of α-proteus, it is hypothesized that the bacterial ancestor of mitochondrial survival contains 3000 to 5000 functional genes, while the mitochondrial primitive ancestor genome contains no more than 1700 functional genes, implying that approximately 1000 to 3000 functional genes were lost during the stage of mitochondrial transition from bacterial symbiont to primitive ancestor^2^. Endogenous genes transfer approximately 2000 functional genes into the host nuclear genome.

In plant cells, gene transfer refers to the transfer of DNA between organelles (mitochondria and chloroplasts) and the nuclear genome, also known as endogenous gene transfer^3^. Because mitochondria are capable of active recombination and exogenous DNA absorption, as well as close contact and fusion, the transfer of endogenous gene sequences in plant mitochondrial genomes is very common. Plant mitochondrial genomes can integrate chloroplast and nuclear genomic origin sequences and other mitochondrial genomic sequences^4–6^. Analysis of the rice mitochondrial genome showed that 13.4% of the sequences were derived from the nuclear genome and 6.3% from other plastid genomes^7^. Numerous integrated mitochondrial fragments in *Arabidopsis* originated from alien genomes, including 16 sequences transferred from other plastids, 41 fragments from nuclear transposons or retrotransposons, and 2 fragments from fungal viruses^8^. The common horizontal transfer genes in mitochondrial genome were *rps11, atp1, nad1B-C*, etc., which occurred not only in the same species, but also in different species^9^. Won and Renner^10^ showed that an intron containing portion of the mitochondrial nad1 gene had been transferred from an angiosperm to the gymnosperm *Gnetum* based on a study of *Gnetum* phylogeny. Phylogenetic analyses provided strong evidence that *rps11* is absent from all 182 core eudicots examined except *Lonicera* and *Betula *^11^. Although the sweet potato organelle genomes are being sequenced, there is no direct evidence of sweet potato nuclear genes or sequences migrating to organelle genomes.

Sweet potato (*Ipomoea batatas* Lam.), belonging to *Convolvulaceae*, is an important economic crop in China^12^. Sweet potato is an asexual reproduction crop, and the analysis of sweet potato organelle genome information is useful for determining genetic information between parents and offspring. Yan et al.^13^ investigated the chloroplast genome of the sweet potato cultivar Xushu18, which had a chloroplast genome of 161,303 bp, relatively conserved compared to other plants. Zou et al.^14^ also confirmed that the chloroplast genomes of sweet potato cultivars were conserved. Mitochondrial genomes in plants are less conservative than chloroplast genomes and are also linked to self-incompatibility in some plants. Understanding sweet potato's evolutionary relationship and compatibility require examining its mitochondrial genome. In this study, we assembled and analyzed the sweet potato chloroplast and mitochondrial genomes, compared organelle genome homology, investigated organelle genome horizontal gene transfer, and confirmed it with PCR sequencing. The findings will be useful in understanding the characteristics of the sweet potato organelle genome and the fertility of sweet potato hybridization.

## Methods

### Plant growth conditions, DNA extraction, and sequencing

The leafy sweet potato 'Fucaishu 18' was used as the test material, and its young shoots and young leaves were collected from the tissue culture seedlings of Fucaishu18 in July 2021, snap-frozen immediately in nitrogen and stored at -80 °C until further processing. The CTAB method was used to isolate DNA from young shoots and leaves^15^. The DNA sample quality was examined with agarose-gel electrophoresis, and the concentration was measured using the Nanodrop instrument (2000c UV–Vis). The third-generation sequencing was implemented on the PromethION. The second-generation sequencing was implemented on the BGISEQ-500 platform.

### Assembly and annotation of the chloroplast genome and mitochondrial genome

The third-generation sequencing reads were aligned to all *Convolvulaceae* mitochondrial genome data of NCBI using Minimap2^16^ and reads with an alignment length greater than 5000 bp were extracted for subsequent assembly. The second-generation sequencing reads were compared to the entire mitochondrial genome using bowtie2^17^, and the aligned reads were used for subsequent assembly. Unicycler was used to assemble the mitochondrial genome using the above-mentioned mitochondrial candidate third- and second-generation reads^18^.

Chloroplast and Mitochondrial gene annotation were performed using GeSeq (https://chlorobox.mpimp-golm.mpg.de) using genomes of the following species as references: *Arabidopsis thaliana* (NC_037304), *Solanum lycopersicum* (NC_035963), *Nicotiana tabacum* (MN651324), *Cuscuta japonica* (MZ240728), *Ipomea bifolra* (MZ240723). The tRNA was annotated using the tRNA scan-SE online website (http://lowelab.ucsc.edu/tRNAscan-SE/). Moreover, the rRNA was annotated using RNAmmer 1.2 Server (http://www.cbs.dtu.dk/services/RNAmmer/) link. The final annotation result is obtained after manual correction. Finally, OGDRAW (https://chlorobox.mpimp-golm.mpg.de/OGDraw.html) software was used to generate a physical genome map^19^.

### Analysis of repeated sequences and RNA editing analysis

The RSCU value (relative synonymous codon usage) of the sweet potato chloroplast genome and mitochondrial genome were statistically analyzed, and the codon bias was analyzed, as previously explained by Sharp et al.^20^. Furthermore, MISA software (http://pgrc.ipk-gatersleben.de/misa/) was used to perform microsatellite scanning analysis on the chloroplast genome and mitochondrial genome sequence of *Ipomoea batatas*. The parameters were set as mononucleotide, dinucleotide, and trinucleotide. The repeat numbers of nucleotides, tetranucleotides, pentanucleotides and hexanucleotides are 10, 6, 5, 5, 5 and 5, respectively^21^. Tandem repeats can be detected using Tandem Repeats Finder v4.04 software, with the default parameters^22^.

The editing sites in *Ipomoea batatas* mitochondrial RNA were discovered using plant mt gene encoding proteins as references. The experiment was carried out using the Plant Predictive RNA Editor (PREP) suite (https://www.hsls.pitt.edu/obrc/index.php) and a cut-off value of 0.2^23^.

### Chloroplast to mitochondrion DNA transformation

DNA migration is common in plants and varies by species. During autophagy, gametogenesis, and fertilization, this phenomenon occurs. Blastn and minimap2 software was used to identify the protein-coding and tRNA genes transferred from chloroplasts to mitochondria. Screening criteria were set as the Identity ≥ 97%, E-value ≤ 1e^−10^, and length ≥ 40. Homology between mitochondrial genome and chloroplast genome was detected by LASTZ version 1.02.00. Furthermore, the synteny map of mitochondria and chloroplasts was drawn by using circlize R package^24^. Sweet potato nuclear genome data comes from the database (https://www.sweetpotao.com/download_genome.html).

The combination of second-generation short fragment sequencing and third-generation long fragment sequencing techniques can ensure the accuracy of the sequence, while PCR sequencing further confirms the horizontal transfer of the sequence. We designed upstream primers from homologous fragment sequences and downstream primers from mitochondrial and chloroplast genome sequences for PCR amplification and Sequencing (Table 1).

**Table1:** Primers for PCR.

Primer names	Sequences
mts21719-F	GGTAGCTTGGAGGATTAAAAGC
mts21719-R	GTCGTGGGCATAAGGGTCTT
chs21719-R	TGATCTTGTGCGGATAGCGG
mts122154-F	TCATATTCGCCCGGAGTTCG
mts122154-R	GCATCCCTAGCGGTACGAAA
chs122154-R	CAACGGAACCGGGGAAAGTA
mts146768-F	AACGCCTCCTAATTTCCGGG
mts146768-R	GCAAAACAAACACGCAAGGC
chs146768-R	AACTGAAGGTACGGAAAGAGAGGG

### Phylogenetic tree construction

The conserved protein-coding genes from mt genomes of *Ipomoea batatas* and 13 other taxa were used for phylogenetic tree construction. The cpDNA and mtDNA were obtained from NCBI, and the conserved protein-coding genes were extracted using the TBtool software^25^, followed by sequence alignment using the mafft software (default parameters –op 1.53,-ep 0)^26^. Then, ML evolutionary tree was selected the Poisson Correction model constructed using FastTree software with Generalized Time-Reversible (GTR) model, Shimodaira–Hasegawa (SH) test, *Nicotiana tabacum* and *Solanum lycopersicum* were designated as the outgroups in this analysis^27^.

### Statement for plant guidelines

All the plant materials in this work comply with the IUCN Policy and the Convention on the Trade in Endangered Species of Wild Fauna and Flora.

### Ethical approval

This article does not contain any studies with human participants or animals performed by any of the authors.

## Results

### The leafy sweet potato cpDNA and mtDNA assembly and annotation

**Figure 1:**
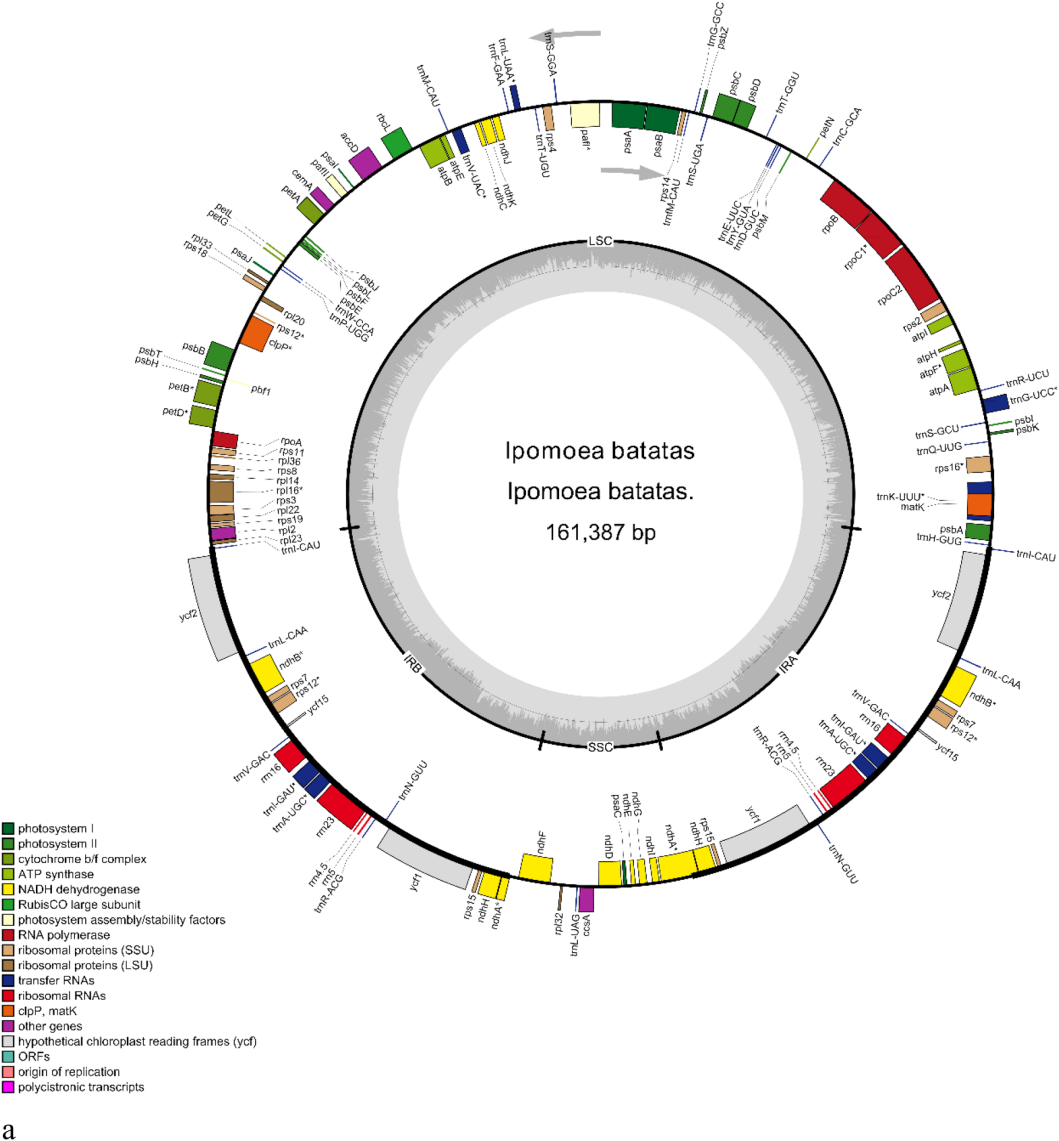

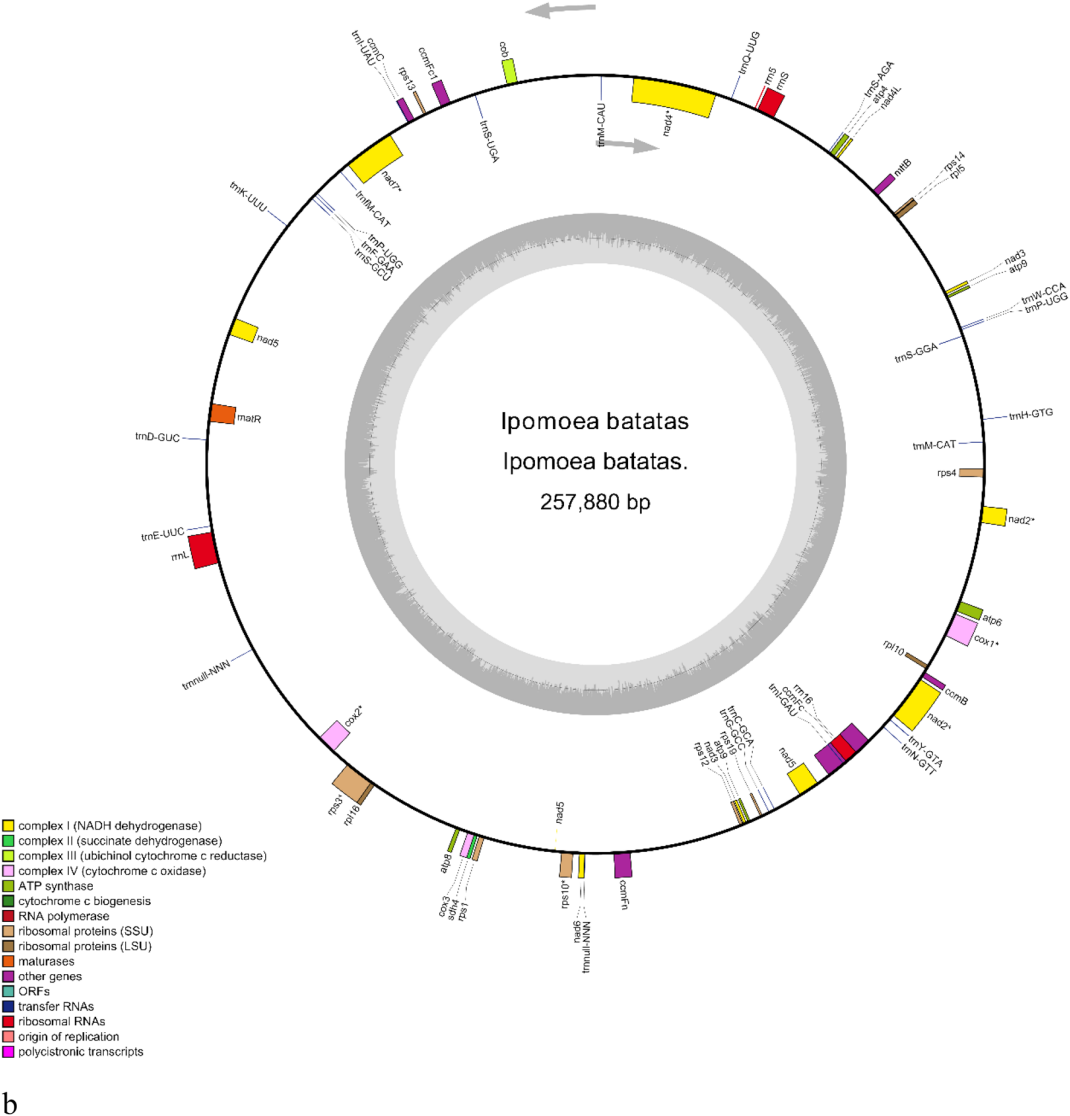
Gene map of the chloroplast genome (**a**) and the mitochondria genome (**b**) of *Ipomoea batatas*. Genes drawn inside the circle are transcribed clockwise, while those drawn outside are transcribed counterclockwise.

The leafy sweet potato full-length cpDNA sequence was 161,387 bp. It was divided into four sections: a large single-copy region (LSC, 87,597 bp), a small single-copy region (SSC, 12,052 bp), and two inverted repeat regions (IRA and IRB, 30,869 bp) (Fig. 1a). Gene annotation results showed that the leafy sweet potato cpDNA had 132 functional genes, including 87 protein-coding genes, 8 rRNA genes and 37 tRNA genes. In the IR region, 18 genes were duplicated, including 7 protein-coding genes (*ycf1, ycf2, ycf15, ndhB, ndhH, rps7,* and *rps15*), 7 tRNA genes (*trnI-CAU, trnL-CAA, trnV-GAC, trnI-GAU, trnA-UGC*), 4 rRNA *(rrn4.5, rrn5, rrn16* and *rrn23*). The total GC content of leafy sweet potato cpDNA was 37.54%, and the AT content was 62.46%.

Statistical analysis revealed that 20 genes in the leafy sweet potato cpDNA contained introns. There were 11 protein-coding genes and 7 tRNA genes with one intron and two protein-coding genes (*pafI* and *clpP*) with two introns (Table S1). There are two copies of *rps12*, each with three exons; the two copies shared the first exon, located in the LSC region, and the other two exons in the IR region. The full-length mtDNA sequence of leafy sweet potato was 269,578 bp. According to the gene annotation results, the leafy sweet potato mtDNA contained 69 functional genes, including 39 protein-coding genes, 6 rRNA genes, and 24 tRNA genes (Fig. 1b). The coding genes included 4 ATP synthase genes, 8 NADH dehydrogenase genes, 5 cytochrome c biogenesis, 3 cytochrome c oxidase, 13 ribosomal proteins, 1 maturase, 1 ubichinol cytochrome c reductase, and 1 transport membrane protein, 1 succinate dehydrogenase and 1 RNA polymerase, of which *nad2, nad4, nad7, cox1, cox2, rps3* and *rps10* contained introns (Table S2). The leafy sweet potato mtDNA had a total GC content of 44.1% and an AT content of 55.9%. A is 27.9%, C is 22.2%, G is 21.9%, and T is 28.0%.

Even if the codons encoding amino acids were the same for different protein structures of organisms, the frequency of use of synonymous codons for amino acids was not equal. In order to analyze the pattern of codon usage in a genome, the set of Relative Synonymous Codon Usage (RSCU) values were computed for each gene.The total length of protein-coding genes in leafy sweet potato mtDNA was 34,710 bp, most of which begin with typical ATG codons. However, *nad4L* and *rps10* begin with ACG, *mttB* and *rps3* begin with TTG, and *cob* begins with GTG (Table S1). There are 32 codons in mtDNA with RSCU values greater than 1.00, the majority of which end with A or T, only three of which end with G (ATG, TTG, TGG), the number of codons encoding leucine (Leu) was the greatest, 1198, accounting for 10.54%; cysteine (Cys) appeared the fewest, 173, accounting for 1.52% (Table S3).

The chloroplast gene sequences (Accession number: OM808940) and mitochondrial gene sequences (Accession number: OM808941) were assembled, annotated, and submitted to GenBank.

### SNP analysis of chloroplast genome in sweet potato varieties

Intraspecific SNP analysis of the chloroplast genome of sweet potato cultivar 'Fucaishu 18' showed that there were 199 SNP sites 118 of which were located in the coding region. The coding region contained *matK, rpoC2, psaB, accD, psbL, rps8, ycf1, ycf2, ndhB, ndhC, ndhE* and *ndhF* genes. Other SNP sites were located in the non-coding region. Compared with other sweet potato cultivars, the chloroplast genome of 'Fucaishu 18' had 121 deletions, of which 37 were SSRs, 146 insertions, of which 66 were SSRs, and 7 long substitutions (Supplementary Dataset File 1).

### Repeat sequences analysis and RNA editing analysis

SSR (Simple Sequence Repeats) was a tandem repeat sequence consisting of 1 to 6 nucleotide repeat units. The single-copy sequences flanking each SSR were generally relatively conserved. The leafy sweet potato organelle genome contained 69 SSR sites, including 54 SSR sites in cpDNA and 15 sites in mtDNA (Table S4).

RNA editing was the insertion, deletion, or substitution of nucleotides in the mRNA produced by gene transcription, resulting in a sequence that is not complementary to the gene's coding sequence, and the amino acid composition of the protein produced by translation differs from the gene sequence information. RNA editing occured in organelles such as mitochondria and chloroplasts, primarily in the mitochondrial genome, and was closely related to organelle function. In nature, mitochondrial transcripts of flowering plants had 300–500 editing sites, whereas chloroplast transcripts had only 30–50 editing sites. According to this study, the mitochondrial genome of sweet potato contained 492 RNA editing sites but only 48 RNA editing sites in the chloroplast genome (Figure S1).

### Organellar phylogenetic relationships

**Figure 2:**
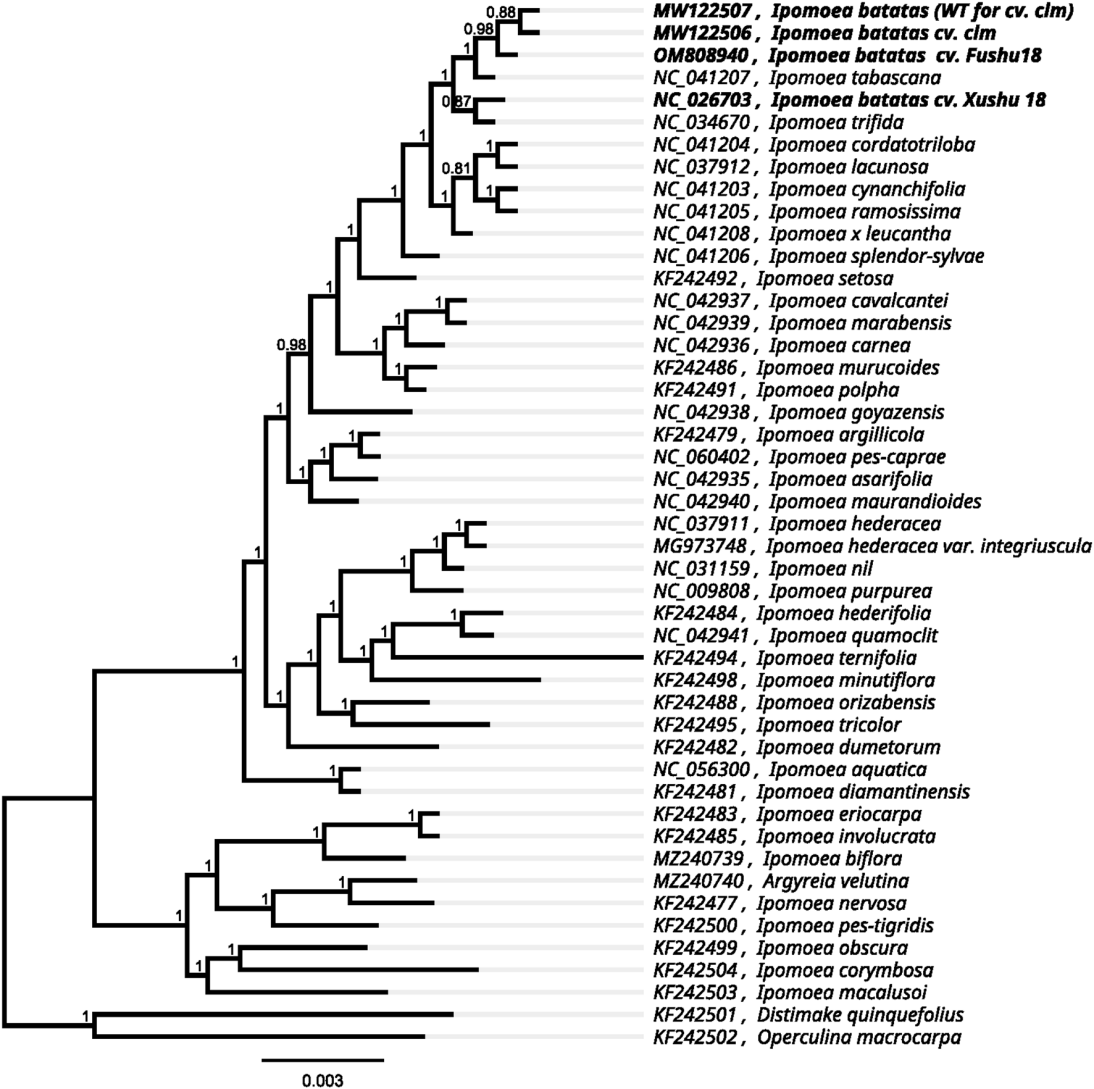
Phylogenetic tree of 42 species of *Ipomoea* based on chloroplast protein-coding genes.

We downloaded 40 *Ipomoea* and two outgroup chloroplast genomes from GenBank (https://www.ncbi.nlm.nih.gov/genome/browse/) to determine *I.batatas'* phylogenetic position. As shown in Fig. 2, all the nodes in the generated tree had 100% bootstrap support values. The phylogenetic tree supported the close relationship between *I.batatas*, *I.tabascana and I.trifida*. Overall, our chloroplast genome analysis findings provided a valuable foundation for future studies of the phylogenetic affinities of *Ipomoea* species.

**Figure 3:**
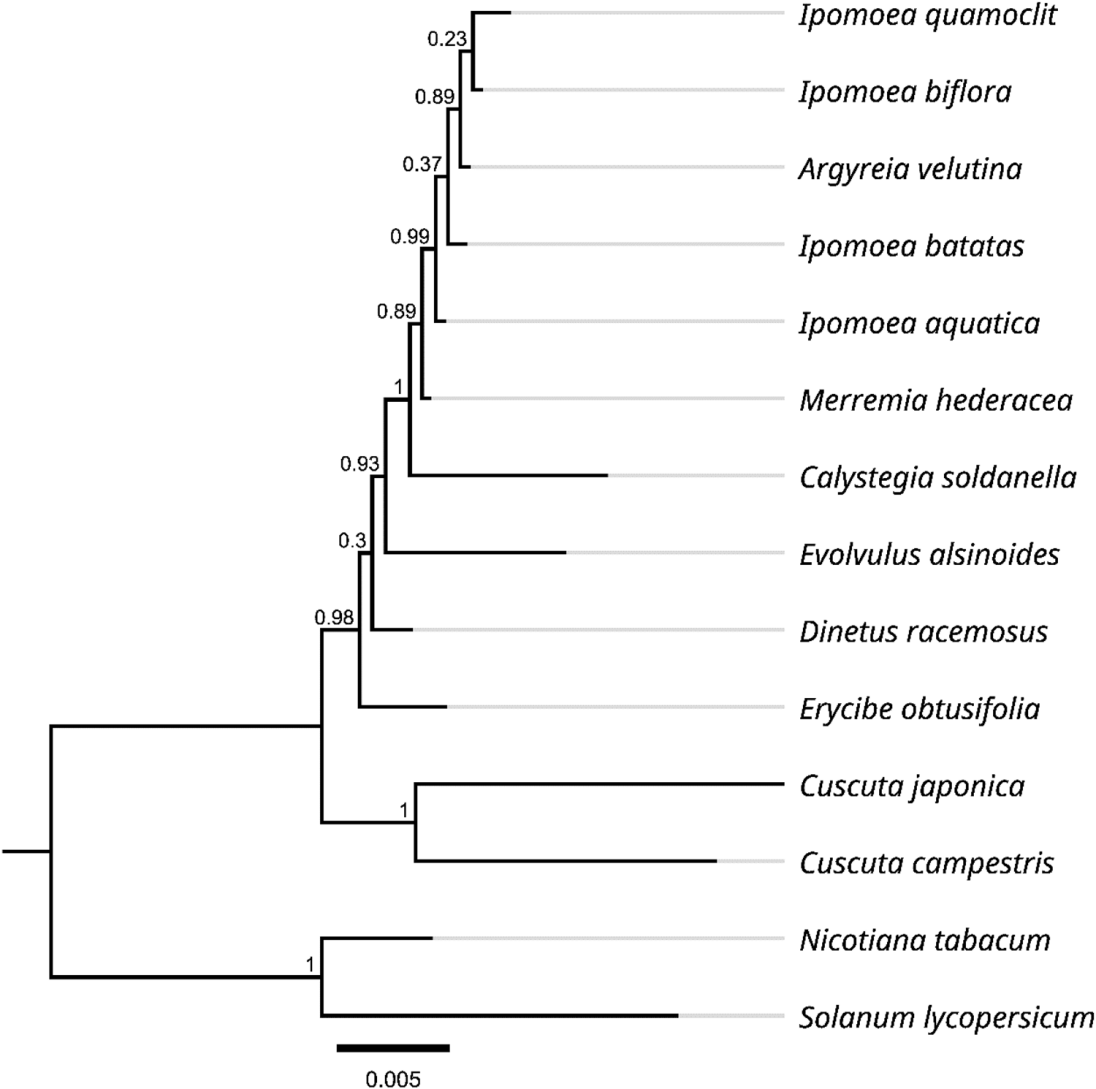
Phylogenetic tree of 14 species based on mitochondria protein-coding genes.

There are few mitochondrial genome data of *Ipomoea* in the NCBI database. We used mitochondrial genome data from several other plants to construct phylogenetic trees. As illustrated in Fig. 3, 3 of the 11 nodes in the generated tree had less than 40% support values. *Argyreia velutina* did not belong to *Ipomoea* but was clustered with other *Ipomoea* plants, indicating that the more mitochondrial genome was need needed for evolutionary analysis.

### Sequence transfer between genomes

#### Length differences of transferred sequences

We evaluated the frequency and pattern of sequence transfer in organelles genomes by having complete and high-quality assemblies for *I. batatas*' nuclear genomes, chloroplast genomes and mitochondrial genomes.

**Figure 4:**
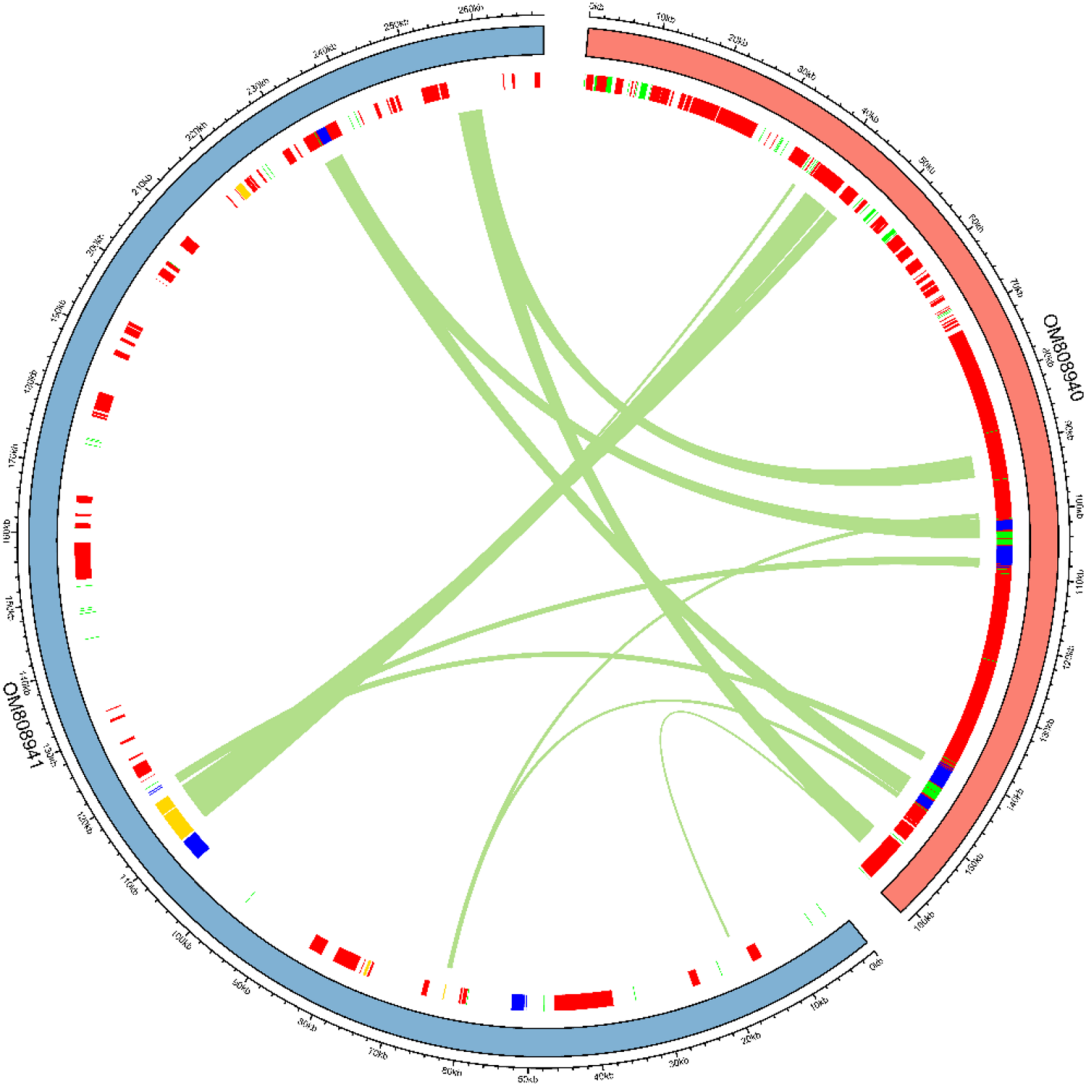
Shared linear mapping of mitochondrial and chloroplast homologous fragments in *Ipomoea batatas.*

The transfer locations were classified into five categories based on the chloroplast genome's sequence content and annotations: exon, intron, rRNA, tRNA, and CDS regions. Reflecting the results from the length of transfers analysis, all transfer location types were predominantly occupied by sequences of chloroplast origin (Fig. 4). Table 2 showed that the length of gene transfer fragments in the mitochondrial genome ranges from 153 to 4013 bp, with only 3 fragments less than 1000 bp occurring in the CDS intervals of *ycf2, ycf15,* and *psbZ*. Sequence transfer of long segments also occurred in the sweet potato organelle genome species. Among them, two fragments of 3581 bp were located in the CDS region of *ycf2*, and the longest fragment of 4013 bp was located in the interval between *psaA* and *psaB* (Table 2).

**Table 2:** Fragments transferred from chloroplasts to mitochondria in *Ipomoea batatas.*

No	Length	mt start	mt end	cp start	cp end	type
1	286	110695	110980	36884	37191	*psbZ* CDS
2	4013	110965	114977	39398	43366	*psaA* CDS
3	1839	114953	116791	43951	45773	*pafI* CDS
4	1304	117584	118887	107389	108687	*23s rRNA*
				140298	141596	*rrn5 rRNA*
5	2911	232693	235603	144964	147860	trnI-GAU exon 1
				101125	104021	trnI-GAU intron 1
6	3581	255852	259432	154802	158172	*ycf2* CDS
				90813	94183	
7	3581	255852	259432	90813	94183	*ycf2* CDS
				154802	158172	
8	2911	232693	235603	101125	104021	trnI-GAU exon 1
				144964	147860	trnI-GAU intron 1
9	1304	117584	118887	140298	141596	*23s rRNA*
				107389	108687	*rrn5 rRNA*
10	574	62966	63539	148170	148755	*ycf15* CDS
				100230	100815	
11	153	16906	17058	157917	158073	*ycf2* CDS

By comparing the mitochondrial genome and nuclear genome of sweet potato, we could find that 171 mitochondrial genome sequences come from the horizontal transfer of nuclear genome (Supplementary Dataset File 2). These sequences covered all the chromosomal genome sequences of sweet potato. Among them, the most 19 sequences were from chromosome 8, accounting for 11. 1%, 18 sequences from chromosome 5, accounting for 10. 5%, and the least were from chromosome 4 and chromosome 14, with only 5 and 6 sequences respectively.

#### PCR Determining gene sequence transfer

**Figure 5:**
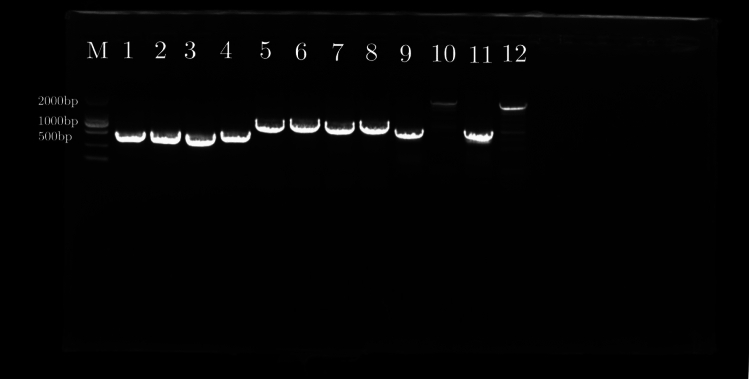
Detection of homology of organelle gene sequences by electrophoresis in *Ipomoea batatas*. M: maker, Lane 1,3: Primer1 used for amplification from mtDNA, Lane 2,4: Primer1 used for amplification from cpDNA, Lane 5,7: Primer2 used for amplification from mtDNA, Lane 6,8: Primer2 used for amplification from cpDNA, Lane 9,11: Primer3 used for amplification from mtDNA, Lane 10,12: Primer3 used for amplification from cpDNA.

We used the same upstream primer but different downstream primers to amplify the homologous sequence by PCR to confirm the gene transfer sequence. The amplified sequence was then sequenced to determine whether it is from the chloroplast or mitochondrial genome (Fig. 5).

The results demonstrated that the 6 pairs of primers could amplify the corresponding bands from sweet potato total DNA. After comparison, it was discovered that 3 were from the sweet potato chloroplast genome sequence and the other 3 were from the sweet potato mitochondrial genome. The results were in line with expectations, indicating that the chloroplast genome and mitochondrial genome of sweet potato. It shows a phenomenon of gene transfer (Supplementary Info File 2).

## Discussion

### Characterization of the *Ipomoea batatas* chloroplast and mitochondrial genome

The traditional method for obtaining plant organelle genomes was to isolate chloroplasts or mitochondria from plant tissues and then extract DNA to obtain a complete genome sequence using first-generation sequencing technology, but isolating mitochondria or chloroplasts from plant tissues was extremely cumbersome^28^. Platforms for second-generation sequencing and single-molecule real-time sequencing have high throughput and can generate a large amount of data with high accuracy. Plant organelle genomes can be obtained quickly using the genomes of closely related species as a reference. In this study, we sequenced and assembled sweet potato's complete chloroplast and mitochondrial genomes using second and third-generation sequencing platforms. The total length of the leafy sweet potato cpDNA sequence was 161,387 bp. The chloroplast genome size of sweet potato was found to be in the range of 161,303 bp to 161,429 bp from NCBI, demonstrating the highly conserved nature of the chloroplast genome^13,14^. Similarly, the full-length mtDNA sequence of leafy sweet potato was 269,578 bp, similar to the genome of the sweet potato variety Jinshan57^29^. The sweet potato chloroplast and mitochondrial genomes have been previously reported and published, and chloroplast genomes only use second-generation sequencing^13,14^, with less accuracy than combining second-generation and third-generation sequencing. Horizontal sequence transfer analysis was not performed on the mitochondrial genome. Plant mitochondrial genome can integrate chloroplast and nuclear genome sequences, and the third-generation of long fragment sequencing can ensure that the integrated sequences were not mistakenly spliced to other sequences^30^.

### Organellar phylogenetic relationship

Complete organellar genomes have been used as foundational markers in determining species phylogenies relationships^31^. Zhang et al.^32^ found that *Ficus* species diverged rapidly during the early to middle Miocene, and *clpP*, *rbcL*, and *ccsA* genes showed positive selection based on the complete chloroplast genome sequences. The chloroplast genome sequences were used to study systematic evolutionary relationships between species of *Swertia* L. It was found that *rpoC1*, *ccsA*, *ndhI*, *ndhA*, and *rps15* protein-coding genes had large variations^33^. The complete chloroplast genomes of *Paraboea (Gesneriaceae)* plants were used to study their phylogeny and adaptive evolution^34^. The complete chloroplast genomes of *Ipomoea* were used to construct a phylogenetic tree in our study. The phylogenetic tree support value was nearly 100% due to the conserved monocyclic structure of the plant chloroplast genome. As a result, the entire chloroplast genome sequence must be used to study plant evolution.

Plant mitochondrial genome size, structure, and sequence content lability across species have severely limited its use in taxonomic studies. Plant mtDNA is much larger than other eukaryotic mtDNA and evolves very rapidly in structure^35,36^. In bilaterians, animals have evolved a relatively compact mitochondrial genome between 11 and 50 Kb in length with a highly conserved gene content. In contrast, plants have large mitochondrial genomes ranging from 66 Kb to 11.3 Mb with large intergenic repetitions prone to recombination^37^. Therefore, in our study, the value obtained using the mitochondrial genome for phylogenetic analysis is not as high as that of the chloroplast genome.

### Sequence transfer between genomes

In plants, DNA within a single cell may be part of the mitochondrial genome, chloroplast genome, or nuclear genome. The presence of these three genomes within a cell provides three possible types of intracellular HGT: between mitochondria and the nucleus, between chloroplasts and the nucleus, and between mitochondria and chloroplasts^4^. In this study, we mainly discussed the HGT between mitochondrial genome and chloroplast genome and nuclear genome in sweet potato. The average read length of the second-generation sequencing was 150 bp, and total read length was 35,906,864 bp. The average short reading length was easy to cause the misjudgment of HGT. The average read length of third-generation was 7,521 bp, the maximum read length was 467,801, and the total read length was 11,504,367 bp. Third-generation sequencing as a supplement allowed the data to better analysis HGT.

Today, HGT into mitochondria occurring between distantly related higher plant species is a well-known phenomenon, and Woloszynska et al.^38^ was the first to demonstrate horizontal transfer of DNA sequences from chloroplasts to mitochondria using a fragment of chloroplast *trnA* gene intron, named *pvs-trnA*. He found that the *pvs-trnA* sequence which contains only 190 bp was identified in only three species of the genus *Phaseolus* and it differs from the chloroplast *trnA* sequence of *Phaseolus* genus in as many as 10 positions and is most similar to the chloroplast genes of *Philodendron scandens* and *Magnolia grandiflora*. In this study, we found that there may be 10 fragments of chloroplast-to-mitochondria transfer in sweet potato by BLAST search, *psbZ*, *23s rRNA* and trnI-GAU exon 1 were present in the chloroplast and mitochondrial genomes using a PCR approach and sequencing. In addition, we found that *ycf2* appeared three times fragment transfer in the chloroplast and mitochondrial genomes of sweet potato. *ycf2* was a relatively large gene in the chloroplast genome, which encoding a long protein, and why fragment transfer occurs more frequently in sweet potato organelle genomes was worth thinking.

The mitochondrial genomes in seed plants were usually structurally conserved and remain 16 kb in size^39^. Integrating foreign DNA into seed plants' mitochondrial genomes may be closely related to their recombination activity. There are few mitochondrial sequences migrating into the chloroplast genome^40^. In contrast, the transfer of chloroplast genomic DNA into mitochondrial genomes was more common and found in plants 3 million years ago^41^. *Cinnamomum camhora* mitochondrial genome contained a 128 kb plastid genome sequence. Most of the plastid fragments that migrated into mitochondria were genes, pseudogenes, and intergenic regions, with the functional genes mostly being tRNA genes and the pseudogenes mostly being genes encoding important chloroplast proteins^42^. In this study, chloroplast-to-mitochondria transfer mainly involved in seven fragments of gene CDS, four fragments of rRNA and two fragments of tRNA. *psbZ*, *psaA* and *pafI* were important genes encoding proteins in chloroplasts, and were possibly transferred from chloroplasts to mitochondrial genes.

### Supplementary Information

Supplementary Information 1.

Supplementary Information 2.

Supplementary Information 3.

Supplementary Information 4.

## Data Availability

The datasets generated or analyzed during the current study are available in the NCBI repository, https://www.ncbi.nlm.nih.gov/.
