# Supplementary material for: Comparative analysis of chloroplast and mitochondrial genomes of sweet potato provides evidence of gene transfer
Source: Sci Rep. 2024 Feb 24;14:4547. doi: 10.1038/s41598-024-55150-1 (PMC10894244; doi:10.1038/s41598-024-55150-1)
Supplement: Supplementary file 3 — Supplementary Information 3. [file 41598_2024_55150_MOESM3_ESM.pdf]

Table S1 List of genes found in Ipomoea batatas chloroplast genome

Category for gene	Group of gene	Name of gene
Photosynthesis gene	Photosystem I gene	*psaA，psaB，psaC，psaI，psaJ*
	Photosystem II gene	*psbA，psbB，psbC，psbD，psbE，psbF，psbH，psbI， psbJ，psbK，psbL，psbM，psbN，psbT，psbZ*
	Cytochrome b/f complex	*petN，petA，psbF，psbE，petG，petB*[a]*，petD*[a]*，petL*
	ATP synthase gene	*atpA，atpB，atpE，atpFa，atpH，atpI*
	NADH dehydrogenase gene	*ndhA*[a]*，ndhB*[a,c]*，ndhC，ndhD，ndhE，ndhF，ndhG， ndhH，ndhI，ndhJ，ndhK*
	Rubis CO large subunit gene	*rbcL*
Self-replication gene	RNA polymerase gene	*rpoA，rpoB，rpoC1*[a]*，rpoC2*
	Ribosomal proteins (SSU) gene	*rps2，rps3，rps4，rps7*[c]*，rps8，rps11，rps12*[b, c]*，rps14*, rps15，*rps16*[a]*，rps18，rps19*[c]
	Ribosomal proteins (LSU) gene	*rpl2, rpl14, rpl16a, rpl20, rpl22, rpl23*[c]*, rpl32, rpl33, rpl36*
	Ribosomal RNAs gene	*rrn4.5*[c]*, rrn5*[c]*, rrn16*[c]*, rrn23*[c]

	Transfer RNAs gene	*trnA-UGC*[a,c]， *trnC-GCA*， *trnD-GUC*， *trnE-UUC*， *trnfM-CAU*， ，*trnF-GAA*， *trnG-GCC*， *trnG-UCC*[a]， *trnH-GUG*， *trnI-CAU*， ，*trnI-GAU*[a,c]， *trnK-UUU*[a]， *trnL-CAA*， *trnL-UAA*， *trnL-UAG*， ，*trnM-CAU*， *trnN-GUU*， *trnP-UGG*， *trnQ-UUG*， *trnR-ACG*， ，*trnR-UCU*， *trnS-GCU*， *trnS-GGA*， *trnS-UGA*， *trnT-GGU*， ，*trnT-UGU*， *trnV-GAC*， *trnV-UAC*[a]， *trnW-CCA*， *trnY-GUA*
Other genes	Translational initiation factor gene	*infA*
	Maturase K gene	*matK*
	Subunit of acetyl-Co A gene	*accD*
	Envelop membrane protein gene	*cemA*
	c-type cytochrome synthesis gene	*ccsA*
	Protease gene	*clpP*[b]
	Hypothetical chloroplast reading frames（ycf）	*ycf1*[c] ，*ycf2*[c] ，*ycf15*，*pafI*[b]，*pafII*

Note：a and b present an intron and two introns in protein-coding genes，respectively. c present two copies of genes.

Table S2 List of genes found in leafy sweet potato mitochondria genome

Group of genes	Gene name

ATP synthase	*atp1, atp4, atp6, atp8, atp9*
NADH dehydrogenase	*nad1, nad2*, nad3, nad4L, nad5, nad6, nad7*, nad4**
Cytochrome c biogenesis	*ccmB, ccmC, ccmFc1, ccmFc2, ccmFn*
Maturases	*matR*
Ubichinol cytochrome c reductase	*cob*
Cytochrome c oxidase	*cox1*, cox2*, cox3*
Transport membrane protein	*mttB*
Ribosomal proteins (LSU)	*rpl5, rpl10, rpl14, rpl16, rpl36, rps1, rps3*, rps4, rps10*, rps12, rps13, rps14, rps19*
Succinate dehydrogenase	*sdh4*
RNA polymerase	*RNA polymerase*
rrnA	*rrn4.5, rrnS, rrn5×2, rrnL, rrn16*
trnA	*trnM-CAT, trnH-GTG, trnS-GGA, trnP-UGG, trnW-CCA, trnS-AGA, trnQ-UUG, trnM-CAU, trnS-UGA, trnI-UAU, trnfM-CAT, trnP-UGG, trnF-GAA, trnS-GCU, trnK-UUU, trnD-GUC, trnE-UUC, trnG-GCC, trnC-GCA, trnI-GAU, trnN-GTT, trnY-GTA, trnnull-NNN, trnnull-NNN*

Note: *means the gene had intron.

Table S3 RSCU of amino acids in leafy sweet potato chloroplast genome

Amino acid	Codon	RSCU	Number	Amino acid	Codon	RSCU	Number
Ala（A）	GCA	0.962	165	Lys（K）	AAA	1.163	311
	GCC	0.933	160		AAG	0.837	224
	GCG	0.507	87	Met（M）	ACG	0.026	2

	GCT	1.598	274		ATG	3.936	306
Arg（R）	AGA	1.512	187		GTG	0.013	1
	AGG	0.752	93		TTG	0.026	2
	CGA	1.286	159	Phe（F）	TTC	0.835	316
	CGC	0.526	65		TTT	1.165	441
	CGG	0.687	85	Pro（P）	CCA	1.100	168
	CGT	1.237	153		CCC	0.714	109
Asn（N）	AAC	0.637	127		CCG	0.714	109
	AAT	1.363	272		CCT	1.473	225
Asp（D）	GAC	0.603	113	Ser（S）	AGC	0.575	100
	GAT	1.397	262		AGT	1.103	192
Cys（C）	TGC	0.728	63		TCA	1.115	194
	TGT	1.272	110		TCC	0.931	162
Gln（Q）	CAA	1.481	228		TCG	0.816	142
	CAG	0.519	80		TCT	1.460	254
Glu（E）	GAA	1.308	323	Terminator	TAA	1.425	19
	GAG	0.692	171		TAG	0.375	5
Gly（G）	GGA	1.475	274		TGA	1.200	16
	GGC	0.506	94	Thr（T）	ACA	1.013	140
	GGG	0.678	126		ACC	1.063	147
	GGT	1.341	249		ACG	0.571	79
His（H）	CAC	0.511	72		ACT	1.353	187
	CAT	1.489	210	Trp（W）	TGG	1.000	165
Ile（I）	ATA	0.885	264	Tyr（Y）	TAC	0.507	96
	ATC	0.801	239		TAT	1.493	283
	ATT	1.314	392	Val（V）	GTA	1.195	202

Leu（L）	CTA	0.876	175		GTC	0.675	114
	CTC	0.656	131		GTG	0.775	131
	CTG	0.521	104		GTT	1.355	229
	CTT	1.287	257				
	TTA	1.457	291				
	TTG	1.202	240				

Table S4 Summary of the SSR distribution in leafy sweet potato

SSR from cpDNA										SSR from mtDNA				
No.	SSR	Size	SSR start	SSR end	No.	SSR	Size	SSR start	SSR end	No.	SSR	Size	SSR start	SSR end
1	(T)13	13	174	186	28	(GGA)4	12	59833	59844	55	(A)10	10	12505	12514
2	(A)11	11	1499	1509	29	(T)10	10	61307	61316	56	(T)10	10	22150	22159
3	(A)12	12	1627	1638	30	(TA)6	12	63682	63693	57	(T)10	10	35689	35698
4	(TTGT)3	12	6482	6493	31	(A)12	12	65204	65215	58	(A)10	10	37087	37096
5	(A)15	15	7849	7863	32	(T)10	10	68002	68011	59	(CT)6	12	38725	38736
6	(A)11	11	8103	8113	33	(AATA)3	12	68611	68622	60	(A)11	11	47487	47497
7	(AATA)3	12	9787	9798	34	(TTTC)3	12	70897	70908	61	(A)10	10	83859	83868
8	(T)10	10	9974	9983	35	(A)13	13	71118	71130	62	(T)10	10	96040	96049
9	(T)10	10	10322	10331	36	(T)10	10	71636	71645	63	(A)10	10	110673	110682
10	(T)10	10	12680	12689	37	(T)11	11	72788	72798	64	(TA)6	12	115311	115322
11	(T)11	11	13335	13345	38	(AGA)4	12	73321	73332	65	(T)10	10	120722	120731
12	(CAAT)3	12	13896	13907	39	(T)10	10	73678	73687	66	(T)10	10	152487	152496
13	(A)10	10	15855	15864	40	(A)11	11	74034	74044	67	(A)11	11	226768	226778
14	(T)13	13	18901	18913	41	(T)11	11	77369	77379	68	(A)10	10	249692	249701
15	(T)10	10	26702	26711	42	(AAAT)3	12	78552	78563	69	(AT)9	18	265232	265249

16	(A)10	10	28254	28263	43	(AATCAA)3	18	88064	88081
17	(T)13	13	30193	30205	44	(TATC)3	12	93626	93637
18	(TCAA)3	12	30875	30886	45	(TTCTA)4	20	100375	100394
19	(T)10	10	31059	31068	46	(A)12	12	110849	110860
20	(TTC)4	12	36291	36302	47	(A)10	10	111260	111269
21	(T)10	10	36505	36514	48	(ATAG)3	12	121006	121017
22	(A)13	13	37715	37727	49	(T)10...(A)10	64	130241	130304
23	(A)11	11	47936	47946	50	(T)10	10	137716	137725
24	(T)10...(T)13	71	48444	48514	51	(T)12	12	138125	138136
25	(GAAA)3	12	48969	48980	52	(AATAG)4	20	148589	148608
26	(T)12	12	50231	50242	53	(AGAT)3	12	155347	155358

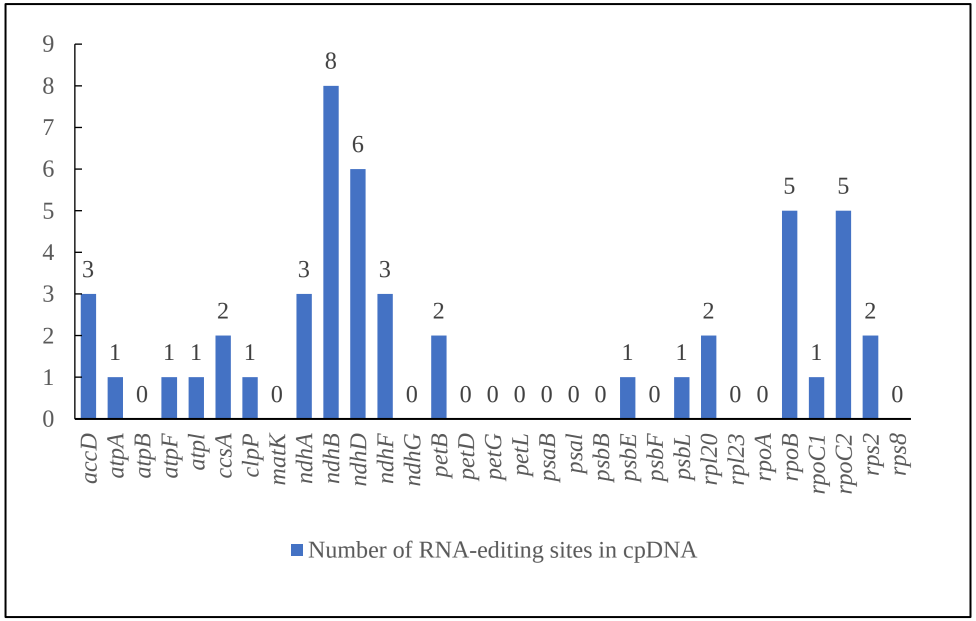

0
1
2
3
4
5
6
7
8
9
3 1 0 1 1 2 1 0 3 8 6 3 0 2 0 0 0 0 0 0 1 0 1 2 0 0 5 1 5 2 0
accD
atpA
atpB
atpF
atpI
ccsA
clpP
matK
ndhA
ndhB
ndhD
ndhF
ndhG
petB
petD
petG
petL
psaB
psaI
psbB
psbE
psbF
psbL
rpl20
rpl23
rpoA
rpoB
rpoC1
rpoC2
rps2
rps8
Number of RNA-editing sites in cpDNA

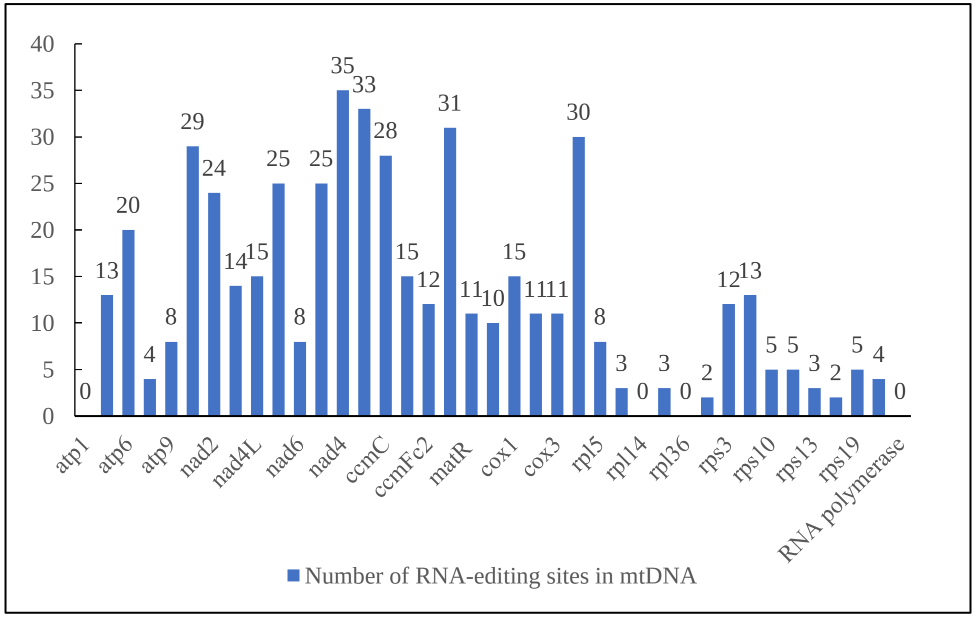

Figure S1 The distribution of RNA-editing sites in *Ipomoea batatas* cpDNA and mtDNA protein-coding genes. The gray bars represent the number of RNA-editing sites of each gene
