# Supplementary material for: Comparative analysis of chloroplast and mitochondrial genomes of sweet potato provides evidence of gene transfer
Source: Sci Rep. 2024 Feb 24;14:4547. doi: 10.1038/s41598-024-55150-1 (PMC10894244; doi:10.1038/s41598-024-55150-1)
Supplement: Supplementary file 4 — Supplementary Information 4. [file 41598_2024_55150_MOESM4_ESM.pdf]

Primer 1 in the mitochondrial genome：

ACGGAGATGCTTATCTTGGCTGTTTTTGCATTAATTGCTACTTCATCAATCTTATTGATTAGTGTAC
CCGTTGTATTTGCTTCTCCGGGGTACCAAAGGCAAGCATGACATCATTATGAACATAAAGTCCC
AAGGTATGGAATCCAAAAAAGAGACTAGCCCAACTTAAATGAGATATGATAGCTTCTTTATGAT
CTAACATTCTTGCCAATACATTATCTTCATTTTGCTCCGGATTGTAATCTCTAATGAAAAATAGAG
CTCCATGAGCAAAAGCTCCTGTCATGATGAATCCTGCGATATATTGGTGATGGGTATATAATGCA
GCTTGAGTAGTAAAGTCTTGTGCTATGAATGCATAAGCAGGTAAAGAGTACATGTGTTGAGCTA
CCAAGGAAGTAATAACCCCTAAAGAAGCTAGAGCTAGGCCTAATTTAAAATGAAGCGAATTATT
GATTGTGTCACAAAGACCCTTTGGCCCACGACATCTTTGCCAAGGAGAAAATGCGGGTTCGATT
CCCGCTATCCGCACCAAAATCAACTCTATCGAAGCTACACCACGTGTTCGAGGTCTCACTTGCTT
ATGCTGATGTTATGGAGGAGCAACAACGACTTCTTGTTCAGAAGGGTGCACATGTTCTCACCTAT
GAAGACCATGATGGAGACTGAATGATGGTTCTTATATGTACCGTGGGAGCTCTGCCTTGGAAGT
GTGAGGATATTGAAGATTGCGTTGACAGATCAATGCTAGCTATCAAGCTTATTCGTGCAGTGGA
CTCTATCTTTCTTTCTTCTTATTATATATCTAGTGTATGAACAGAGAGTTTGCTGACCAGAGGGTA
CATGTGATGCTGAGGCTATACTACCATCGCAAAATATACTGAAGAATAGTCGACCCTGATATCTT
CTTCTCAATGTGACATTTGGGATGTTTTTATGCTTGATACTTTCACCGGCACGTCTTGCACAACGT
CCTGTATGTATTGTATGGGAGCTGAGT

Primer 1 in chloroplast genome：

GTGGTTTGCTTTCATTGGCTGTTTTTGCTTAATTGCTACTTCATCAATCTTATTGATTATGTACCCG
TTGTATTTGCTTCTCCTGAGGGCTGGTCAAGTAACAAAAATGTTGTATTTTCCGGTACCTCATTAT
GGATTGGATTAGTCTTTCTGGTGGGTATACTTAATTCTCTCATCTCTTGACCCTATTCGTCCCAAA
CTCGACAGACCCCCCGAATTTTTCTCGGTTGTGAGACACCTTCAAAATTCATATTCATCTCCGTCT
CCCTCAAAAAAATCCAAATAAATTTCAAATCAAGAAAACCAAAAAAAAGAAAAGGGGGTCAAA
CTTCTTGAATGAAAAAATAAATTGGCATCGTCCTGAAGAGAGTCTTTGGTCCGGCACTGCACAA
ATAGGATCCGGGTATATATCATATATGTGTGTACATATTATGTACATGTGTCAATAACGAAAAAT
TGCGGATATGGTCGAATGGTAAAATTTCTCTTTGCCAAGGAGAAAATGCGGGTTCGATTCCCGC
TATCCGACCAAATCAATTCTATCGAAGCTTCACCACGTGTTCGAGGTCTCCATCGCTTATGCTGA
TGTTATGGAGTACCAACAACGAGTTCCTGTCCAGAATGCTGCACATGTTCTCACCTATGAAGAC
CATGATGGAGACTGAATGATGGTTGGGGATGTACCGTGGGAGCTCTTCCTTGGAAGTGTGAGG
ATATTGAAGATTGCGTTGTAGATCAATGCTAGCTCTCAAGCTTATTCGTGCAGTGGACTCTACAT
TTCTTTCTTCTTTTTATATATAAGTTTATTAACAGAGAGTTTGCTGAACAGAGTGTACCTGAGATG
CTGACGACTATACTAACCGTCGCAAGACACACTGAAGAAAAAGTCGACCTTGATAGCCTTCCCA
CCAATGTTACATTTTGATATTTGTTTGTTTGATACTATCACCGGCACGTCATTGCCACAAGTCCTG
TATGTATTGTATGGAGCTGGAGTTCA

Primer 2 in the mitochondrial genome：

CGAAAAGGTATCCCTGCCCCTCACGTCAATCCCACGAGCCTCTTATCCATTCTCATTGAATCACA
GCGGGGGGGCAAATCCAACTAGAAAAACTCACATTGGACTTAGGGATAATCAGGCTCGAACTG
ATGACTTCCACCACGTCAAGGTTTAAAATTTCGAAGAAGCTCTTAGTTTTATACTTATTTCCCGCA
CTTCTTCAGTAAATTTTCCTACTTTATTATGCCGATTCCTCTCTCCGTATATTTTCTGTTAGAAGGA
AGGCATCGTGATGGTTGTCTATTATTCCTAGCATCATATCTGCGAACACGAACATCAAACTGGAA
ATTTTCACTAAGAGTGAGTGTACCACAAGACAAATCATTTGGTTCATTCTTTGACTGCTCCGCTC
CCCCCAAAAAAAACCAACCCTGTAGCGCTAGCGCTTCGCGTTCTTTCTATTCCATCCCATTCCAT

TCCGGGATAGACGGCTAATACTAATCAAGACAAAAGGTGAAGTAGTCGTCGTCTGACCAATCG
GCAGACCGCCCGTGCCCGTCCATTTTTTCTCGCCCTAAATGGAATGGCTCTCTTAGTTACGCTGC
GCCCCGACCCGAGTCCCCACGTCCGCTTTTATCCGCTCGAAACCCAATAAGTTGGCTTTGCCAA
CACAACATTAGGGCCGTCTCCTTCATTCTATGCTGACCCCGGCCCGGGCTGGCTTTTTGGGAAG
CCCGTTCCCACCGCGCTCACGGCCCGGCTGGCCTGCCAGCGGTAGTGGGAATTCTCCCGTTCC
CTGGTCAAAGACTTGGTTGGATGCGGGATCTACTCCACGAGGAGCGGTACGGACGTAGATGAT
ATCATCACGACCCCTCTTTTCGACGTTGGGGGGAAGCACAGAT

Primer 2 in chloroplast genome：
TCCCAAAAGCATCCTGCCCCTCACGTCAATCCCACGAGCCTCTTATCCATTCTCATTGAATCACA
GCGGGGGGGCAAATCCAACTAGAAAAACTCACATTGGACTTAGGGATAATCAGGCTCGAACTG
ATGACTTCCACCACGTCAAGGTGACACTCTACCGCTGAGTTATATCCCTTCCCTGCCCCCATCGA
GAAATTGAACTGACTAATCCTAAGTCAAAGGGTCGAGAAACTCAACGCCACTATCACTATTTTT
GAACAACTTGGAGCCAGGCCTTCTTTTCGCACTATTACGGATATGAAAATAATGGTCAAAATCG
GATTCAATTGTCAACTGCCCCTATCGGAAATAGGATTGACTACCGATTCCGAACCGAAGGAACT
GGAGTTACATCTCTTTTGCACTCAAGAGTTCTTATGTGTTTCCACGCCCCTTTGAGACCCCGAAA
AATGGACAAATTCCTTTTCTTAGGAACACATACAAGATTCGGCACTACAAAAAGGATAATGGTA
ACCCCACCATTAACTACTTCATTTATGAATTTCATAGTAATAGAAATACATGTCCTGCCGAGACA
GAATTTGTAACTTGCTATCCTCTTGCCTAGCAGGCAAAGATTGACCTCCGTGGAAAGGATGATTC
ATTCGGATCGACATGAGAGGCCAACTACATTGCCAGAATCCATGTTGTATATTTGAAAGAGGTT
GACCTCCTTGCTTCTCTCCTGGTACACTTCTCTTCCCGCCGAGCCCCCTTTCTCCTCGGTCCACAG
AGACAAAATGTAGGACTGGTGCCAACAGTTCATCACGGAAGAAAGGACTCACTAAGTCGGGAT
CACTAACTAATACTAATCTAATGAATACTAATCTAATATCTAATAGAATAGAATAGAATAGAAAA
GAACTGTCTTTTCTGTTATCTC

Primer 3 in the mitochondrial genome：
AGATAGATACGATTCGATGGTTATACAGGTATCCACAGTACGAACGAGATGGATGCTTGTTGTC
CCAACCATTCTTTCAATCCCGAGCCCGATAAGGAAGGGGGTAATTTAGAACAAAGTTTTCGTGT
TGTTTATTCCTAGGTGTAGTGATTATTCCCCTATGCTGCTTATTGGCACTAATACAATAGGATTTA
CCCGTAATACAGAACCTCTAGGTGTAACCTTCCGCTCAATACTAGAATCGATAAATGAAACATA
ACATTTGAGGTTGCATTAATCGAGGATACACGACAGAAGGAATTGTTCTATTTCCAAACTTCACC
TTCAACTTGAAAATTGGATTTTTTTTCAATAATTTGGCGTGTCTTTCTCGTAAGACTGAGAGAAAT
GACTAAATATTAAATAAAATATTCTAAAGTAAACTAAGAATCAAATCGCACCATCTCTGTAATAG
GTAAATGCCTCTTTTTCTCCTGAAGTTGTCGGAATTATTCGTAATAAGCTAAAGATTATAAAAAAC
AAAACAAGCAACGGCTTTCGCGTCAGCCCTTGCTTGTTTCCAGCCGTTGCTTGTTGGTTGGTGCT
AATCTAAGGTAGCTGCAGAAGAAAGAAAAGAAGACGGTAAAAGTACATCGGCATCAATGCTGC
TGCCCAGCTTCTCCTCGATCCGGGCCTTGGG

Primer 3 in chloroplast genome：
AAGTCGATCCCGCTTCGATGGTTATACAGGTATCCACAGTACGAACGAGATGGATGCTTGTTGT
CCCAACCATTCTTTCAATCCCAAGCCCGATAAGGAGAGGGTAATTTAGAACAAAGTTTTCGTGTT
GTTGATTCCTAGGTGTAGTGATTCTTCCCCTATGCTGCTTATTGGCACTAATACAATAGGATTTAC
CCGTTGAACCTCTAGGTGTAACCTTCCGCTCAATACGAGAATCGATAAACGAAACATAGCATTT
GAGGTTACATGAATCGAGGATACACGACAGAAGGAATTGTTCTATTTCCAAACTTCACCTTCAAT

AAGCGTAGATTTTTTTCAATAACTTGGCGTGTCTTTCTCGTAAGACGGAGAGAAATGACTAAAGA
TGAAAGAAATAAAAAACCAAGAATCAAATCGCACCATCTCTGTAATAGGTAAATGCCTCTTTTTC
TCCTGAAGTTGTCGGAATTATTCGTAATAAGATATTGGCTACAATTGAAAAGGTCTTATCAATAA
AATTTCCATTTATCCGCGATCTCGGCATACCATTCTATCATTTTTCTCATTACCTCTCGTGGGAAA
AAGATCCCACAAAGAAAAGAATTGTATAGTACGAAATAACATAAAACATATTTATGAAAAATAT
GAACCTTCTATTCCATATTCCAACTGTTCTTTTTTGGCAGGAATCGAATATTTCAATGTGATAATG
AATGGAGTAGTATAGGAACTATTCCTATAAATTACGGGTTAGAAGAACTCGAGAAATTTTGATTG
AATTGTGACATAAATTAGAAAAAAGATAGAAGAATCTTTCTATGATGAAAATAGACTAACTGCC
AATTTTGTATACATAACAGGTATACACTCTACAATCAAATAGAAAAATGTTTTCTGTGAATTTCTA
TTCAAATCGAGGAATAACGGGTTTGTTGTTGAAAACTCTAAAAGGGAATTTGAGAATTGGTTTGG
TATGGAATCATATATCTAATAGAAT
